# Data on the characterization of native soy globulin by SDS-Page, light scattering and titration

**DOI:** 10.1016/j.dib.2016.10.016

**Published:** 2016-10-25

**Authors:** Nannan Chen, Mouming Zhao, Christophe Chassenieux, Taco Nicolai

**Affiliations:** a College of Light Industry and Food Sciences, South China University of Technology, 510640 Guangzhou, China; b Université du Maine, IMMM UMR-CNRS 6283, Polymères, Colloïdes et Interfaces, 72085 Le Mans cedex 9, France

**Keywords:** Soy protein isolate, Titration, Molar mass

## Abstract

The data presented in this article are related to the research article entitled *Structure of Self-assembled Native Soy Globulin in Aqueous Solution as a Function of the Concentration and the pH* by N. Chen, M. Zhao C. Chassenieux, T. Nicolai (2016) [1]. Please refer to this article for interpretation of the data. The protein composition of soy protein isolate (SPI) was characterized by SDS-Page. The molar mass of native soy globulin aggregates formed at different protein concentrations was determined by light scattering as a function of the waiting time. The dependence of the pH on the net charge density of native soy globulins was determined for solutions containing 5 g/L or 2 g/L SPI.

**Specifications Table**

**Table:** 

Subject area	*Protein*
More specific subject area	*Soy*
Type of data	*Figures*
How data was acquired	*Commercial light scattering equipment ((ALV-CGS3, ALV-Langen)*
	*Automatic titrator (TIM 856, Radiometer Analytical)*
Data format	*Analyzed*
Experimental factors	*SPI was dissolved in Millipore water and centrifuged at 4*×*10*^*4*^ *g for 4 h.*
Experimental features	*Reducing sodium dodecyl sulfate-polyacrylamide gel electrophoresis (SDS-PAGE) was performed with a discontinuous buffered system, using 12% separating gel and 5% stacking gel. Light scattering was done on highly diluted solutions as a function of the scattering wave vector (q). The molar mass was determined from the scattering intensity extrapolated to zero q.*
Data source location	*n/a*
Data accessibility	*Data is with this article*

## 1 Data

**Fig. 1:**
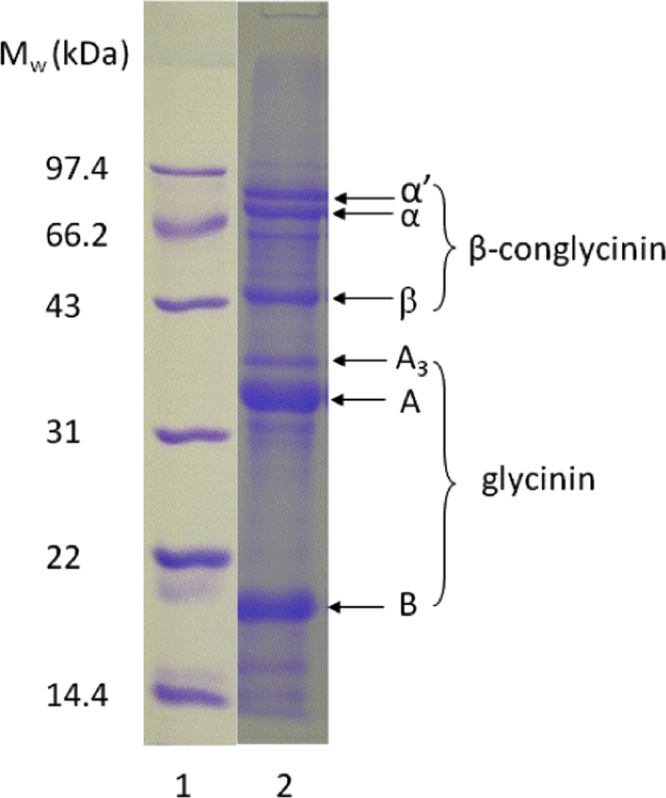
SDS-Page pattern of the soy globulin used for this study. Lane 1 Molecular weight standards. Lane 2 Native soy globulin: α′, α and β represent subunits of β-conglycinin; A, A_3_ and B represent the acid and basic polypeptides of glycinin.

**Fig. 2:**
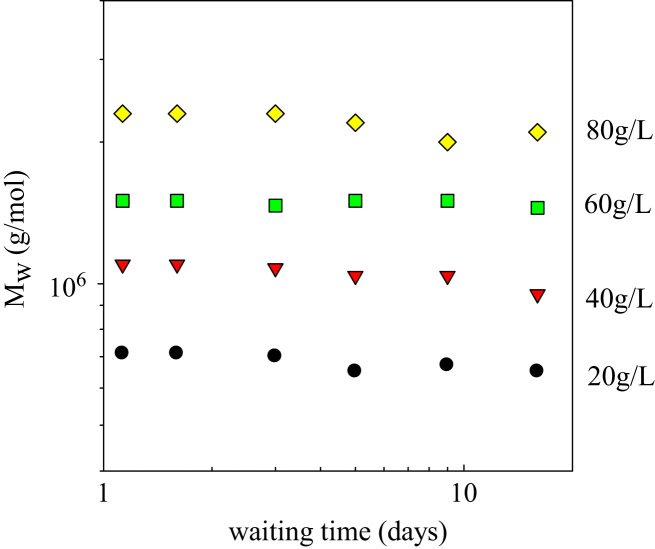
Molar mass of the soy globulin aggregates formed at different protein concentrations as a function of the waiting time before dilution and characterization by light scattering.

**Fig. 3:**
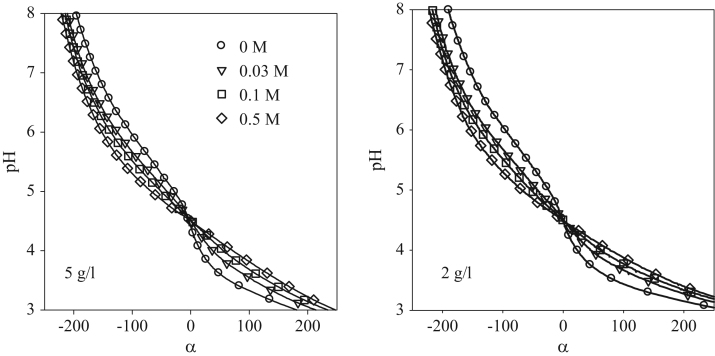
Dependence of the pH on the charge density of native soy globulins at different NaCl concentrations indicated in the figure for solutions containing 5 g/L or 2 g/L SPI. Data at *C*=10 g/L are shown in Fig. 5 of the associated research article [1].

The SDS-Page pattern for SPI is shown in Fig. 1. The dependence of the molar mass of SPI aggregates on the waiting time is shown in Fig. 2 for different protein concentrations. Titration curves of native SPI at *C*=2 g/L and *C*=5 g/L are shown in Fig. 3.

## 2 Experimental design, materials and methods

SPI was obtained from defatted soy flakes as described in Ref. [2]. Aqueous solutions of SPI were prepared as described in Ref. [1]. Reducing sodium dodecyl sulfate-polyacrylamide gel electrophoresis (SDS-PAGE) was performed on a discontinuous buffered system, using 12% separating gel and 5% stacking gel. The molar mass of native SPI aggregates was determined by light scattering as described in Ref. [1]. Solutions were titrated with 0.1 M NaOH and from the amount of added NaOH the net charge density of the protein was calculated as described in Ref. [1].
